# Estrogen-related receptors: novel potential regulators of osteoarthritis pathogenesis

**DOI:** 10.1186/s10020-021-00270-x

**Published:** 2021-01-15

**Authors:** Jinshuo Tang, Tong Liu, Xinggui Wen, Zhongsheng Zhou, Jingtong Yan, Jianpeng Gao, Jianlin Zuo

**Affiliations:** 1 Department of Orthopeadics, China-Japan Union Hospital of Jilin University, Changchun, 130033 Jilin China; 2 Department of Hand Surgery, China-Japan Union Hospital of Jilin University, Changchun, 130033 Jilin China

**Keywords:** Estrogen-related receptors, ERRs, Pathogenesis, Osteoarthritis

## Abstract

Osteoarthritis (OA) is a chronic inflammatory disease that is associated with articular cartilage destruction, subchondral bone alterations, synovitis, and even joint deformity and the loss of joint function. Although current basic research on the pathogenesis of OA has made remarkable progress, our understanding of this disease still needs to be further improved. Recent studies have shown that the estrogen-related receptor (ERR) family members ERRα and ERRγ may play significant roles in the pathogenesis of OA. In this review, we refer to the latest research on ERRs and the pathogenesis of OA, elucidate the structure and physiopathological functions of the ERR orphan nuclear receptor family, and systematically examine the relationship between ERRs and OA at the molecular level. Moreover, we also discuss and predict the capacity of ERRs as potential targets in the clinical treatment of OA.

## Introduction

Osteoarthritis (OA) is the most common joint disease among the elderly population (Glyn-Jones et al. 2015). Approximately one-third of senior citizens over 65 years of age suffer from OA, and the incidence is significantly higher in women than in men. According to epidemiological survey data (Johnson and Hunter 2014), with the aging of the population and the increase in average life expectancy, the incidence and prevalence of OA are soaring. However, the current treatment provided by clinicians for OA patients is still limited to symptom management (Correa and Lietman 2017; DeRogatis et al. 2019), which fails to curb the development of this condition. We now realize that inflammatory cytokines, metalloproteinases, cellular senescence, estrogen and biomechanical imbalances play crucial roles in the progression of OA and can lead to a series of critical pathologic changes (Wang et al. 2017a; Mehana et al. 2019; McCulloch et al. 2017; Watt 2016), such as focal cartilage deficiency, osteophyte formation, subchondral bone remodeling and synovial hyperplasia, in the joints of OA patients (Charlier et al. 2019), but our understanding of the pathogenesis of OA still needs to be improved. In recent years, studies have shown that estrogen-related receptor α (ERRα) and γ (ERRγ) in the estrogen-related receptor (ERR) family may play essential roles in the pathogenesis of OA. In this review, we systematically expounded on the relationship between ERRs and OA at the molecular level by referring to recent research findings.

### Structure of estrogen-related receptors

ERRs are members of the nuclear receptor superfamily and have a tight structural relationship with estrogen receptor α (ERα) and β (ERβ) (Eichner and Giguere 2011). In 1988, two unique nuclear receptors with conserved steroid hormone receptor features were identified by a probe synthesized from a cDNA library, namely, estrogen-related receptors α and β (Giguère et al. 1988). Subsequently, in 1998, the third receptor isoform, estrogen-related receptor γ, was discovered by researchers (Eudy et al. 1998). The ERR family have extensive sequence similarity with the DNA-binding domain (DBD) and ligand-binding domain (LBD) of ERα (Divekar et al. 2016). However, these receptors cannot bind to endogenous estrogen or its derivatives, and so ERRs are also referred to as orphan nuclear receptors (Huss et al. 2015; Tripathi et al. 2020). In human tissue, ERRα has no known splice variants, ERRβ has three splice variants, and ERRγ has two splice variants (Xu et al. 2016). These splice variants signify a significant source of functional diversity in the proteome (Heckler and Riggins 2015); for instance, the expression of the ERRβL splice variant can augment ERα-dependent gene activation (Bombail et al. 2010), and activated ERRβ2 splice variants are potent inhibitors of karyokinesis in breast carcinoma cells, including TNBC (Heckler et al. 2016). However, there are still quite a few limitations in the understanding of these splice variants due to the limited research currently available (Bombail et al. 2010; Bielli et al. 2019).

**Fig. 1:**
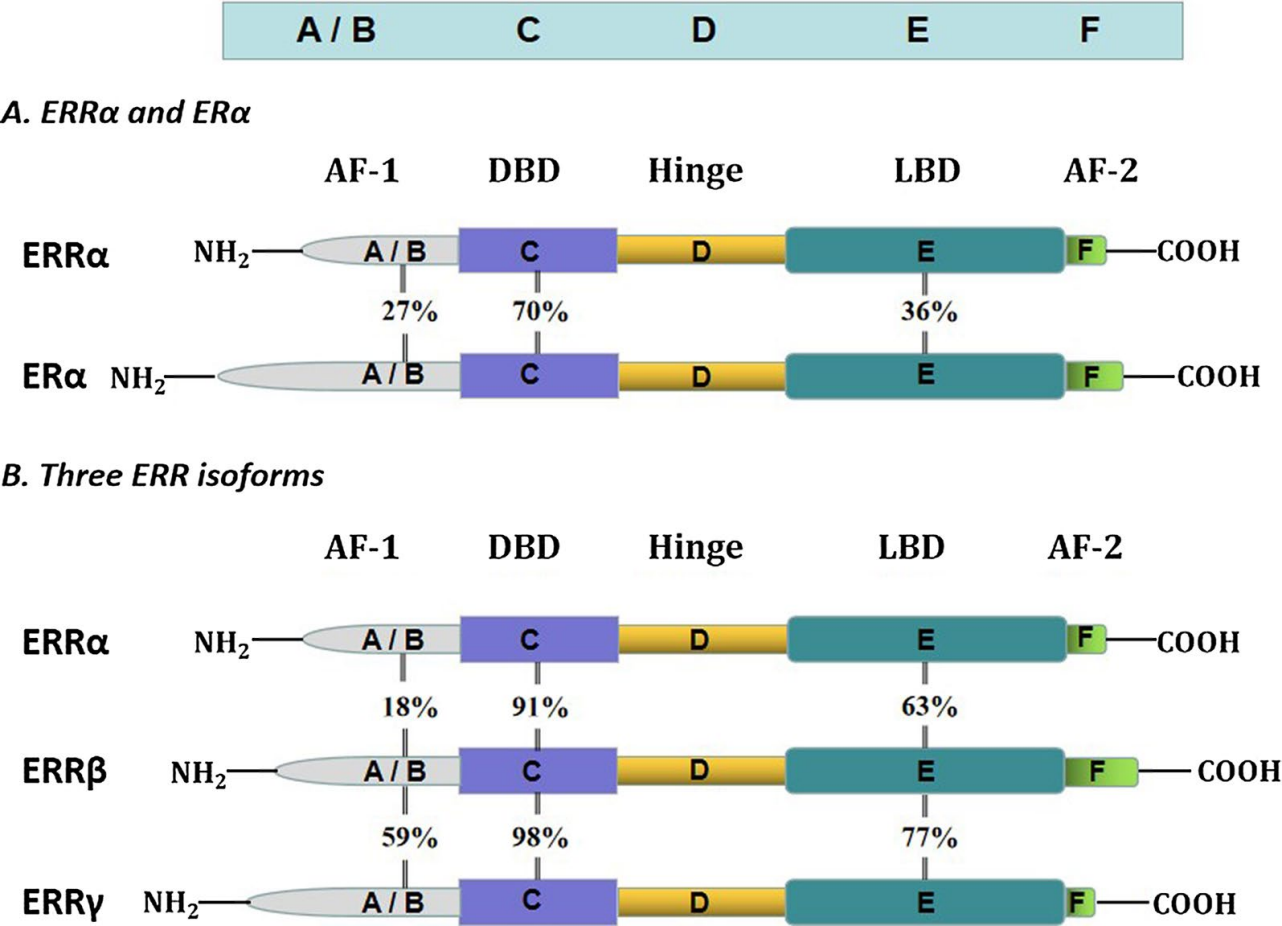
Structure of estrogen receptor α and the three estrogen-related receptors. ERRs are composed of six conserved regions (A/B, C, D, and E/F domains), and the colors symbolize the diverse functions of these domains. The number between the two receptors represents the sequence identity of the same domain in the different receptors

The molecular structure of ERRs is similar to that of other nuclear receptors, and these proteins consist of six conserved regions (A/B, C, D, E/F domains) (Lu et al. 2019) (Fig. 1). The N-terminal region is the A/B domain, also known as activation domain-1 (AF-1), and has the characteristics of ligand-independent transcriptional activation. The A/B domains of ERRs contain conserved motifs that allow their transcriptional activity to be regulated by posttranslational modifications such as phosphorylation and SUMOylation (Vu et al. 2007; Tremblay et al. 2008).

The central C domain of ERRs is referred to as the DBD and contains two highly conserved zinc finger motifs (Saito and Cui 2018), which can bind to a specific DNA sequence (TCAAGGTCA) called the ERR response element (Xia et al. 2019). ERRs can bind with ERR monomers, homodimers or heterodimers consisting of two different ERR isoforms (Mohideen-Abdul et al. 2017; Horard et al. 2004). Because all members of the ERR family have virtually identical C domains, distinct isoforms of ERR can sometimes target the same gene (Casaburi et al. 2018).

The D domain is a flexible hinge region that provides protein flexibility when the dimer is bound to DNA and links the C and E regions (Misra et al. 2017; Helsen and Claessens 2014).

The E/F domain is the ligand-binding domain. The LBDs of ERRs and ERα share 30–40% homology. However, ERRs cannot bind to endogenous estrogen or its derivatives because they lack Cys residues that identify ligands (Gibson and Saunders 2012). The LBD contains a conserved helix motif called activation function-2 (AF-2) (Huss et al. 2015), and this motif is exposed. Three ERRs are constitutively active due to the structure of the E/F domain, which is capable of binding coregulators in the absence of ligand binding (Misawa and Inoue 2015; Liu et al. 2016; Chen et al. 2001). Although the ligand binding pockets of ERRs are smaller than those of ERα, it is difficult to accommodate effective natural ligands (Greschik et al. 2002). However, it is hypothesized that the transcriptional activity of ERRs can still be regulated by certain undiscovered ligands (Gibson and Saunders 2012). A large number of experiments have shown that the use of some synthetic compounds can regulate the function of ERRs. For example, compound LingH2-10 is a novel selective inverse agonist of ERRα (Ning et al. 2017), compound DY181 is also considered to be a selective inverse agonist of ERRβ and has excellent selectivity and effectiveness (Yu et al. 2017), and compound DY40 is a synthetic inverse agonist of ERRγ.

Protein sequence analysis of all members of the ERR family revealed that the DBDs and LBDs of the three ERR isoform have high amino acid sequence homology, suggesting that they may bind to similar ligands and target the same promoter and enhancer elements (Giguère 2002). The ERR family has the highest DBD amino acid sequence identity (91–98%), relatively low sequence homology among LBDs (62–77%), and lowest sequence identity for A/B domains compared to DBDs and LBDs (15–59%).

### Physiological and pathological function of ERRs

The physiological functions of ERRs are complex and varied, and these proteins play crucial roles in controlling the balance of cellular metabolism, general metabolism, growth and development, cancer occurrence, and bone homeostasis (Villena and Kralli 2008; Thouennon et al. 2019; Misra et al. 2016; Zhang et al. 2015; Li et al. 2019a). The expression of ERRs is widespread and is particularly high in tissues with high energy expenditure or vigorous metabolic demands (Festuccia et al. 2018). Among adults, Esrra exhibits the highest expression level, Esrrg exhibits an intermediate level of expression, and Esrrb shows the lowest expression (Likhite et al. 2019). As the site of oxidative metabolism, the metabolic activity of mitochondria is strictly controlled to meet the energy demands of cells under different physiological conditions. The well-known inducers of mitochondrial oxidative metabolism are peroxisome proliferator-activated receptor γ coactivator 1α (PGC1α) and nuclear receptor corepressor 1 (NCOR1) (Brown et al. 2018b; Lima et al. 2018), which are abundantly expressed in high-energy demand tissues such as the heart, skeletal muscle, and brown adipose tissue (BAT). However, both PGC1α and NCOR1 lack DNA binding activity and depend on interactions with transcription factors that directly bind and control downstream target genes. ERRs have been shown to be key transcription factors that regulate mitochondrial oxidative metabolism and induce PGC1α and NCOR1 expression (Fan and Evans 2015). Studies have shown that ERRs can bind and regulate the expression of glycolytic genes (Long et al. 2020), including pivotal enzymes such as phosphofructokinase, hexokinase 2 (HK2), glyceraldehyde phosphate dehydrogenase (GAPDH) and enolase 1 (ENO1), which are crucial components of cellular glucose metabolism (Kida et al. 2015). ERRα is highly expressed in tissues involved in lipid metabolism and energy balance, such as white adipose tissue (WAT) and BAT, the heart and skeletal muscles, which require high oxidative capacity (Audet-Walsh and Giguere 2015). Mice lacking adipose ERRs (ERRαγAd−/−) have reduced oxidative and thermogenic capacity, and when exposed to a low-temperature environment, they rapidly become hypothermic (Brown et al. 2018a). The thermogenesis of BAT depends on the level and activity of mitochondrial uncoupling protein 1 (UCP1) (Oelkrug et al. 2015). Epinephrine stimulates BAT cells to activate UCP1-mediated thermogenesis (Porter 2017), which also stimulates UCP1 gene expression. Recent studies have shown that ERR isoforms function in BAT in a highly complementary manner to control mitochondrial biogenesis and cellular oxidative capacity. There are defects in the transcription and metabolic reaction of BAT lacking all ERRs to adrenaline-stimulated UCP1 (Gantner et al. 2016), resulting in a significant decrease in mitochondrial content and oxidative capacity. ERRs are vital effectors of adrenaline-stimulated BAT transcriptional reprogramming.

The high expression of ERRα is related to the poor prognosis of numerous malignancies and can promote the invasive characteristics of a variety of cancers (Tribollet et al. 2016). In estrogen receptor-negative (ER−) breast cancer cells, ERRα acts as an activating transcription factor. ERRα overexpression increases the growth of breast cancer cells in the mammary gland, as well as the expression of vascular endothelial growth factor (VEGF) (Misawa and Inoue 2015; Fradet et al. 2011). In triple-negative breast cancer (TNBC), ERRα can also regulate the expression of genes needed for cancer cell metabolism, enhancing the ability of breast cancer cells to use lactic acid as a metabolic substrate (Park et al. 2016). Furthermore, ERRα enhances breast cancer resistance to certain anticarcinogens by regulating mitochondrial metabolic adaptation (Li et al. 2020). The combined administration of an ERRα inhibitor and rapamycin to ER− breast carcinoma cells can synergistically suppress the proliferation of tumor cells (Berman et al. 2017). In contrast, ERRα expression is upregulated in urinary bladder carcinoma. After inhibiting the expression of ERRα, the growth, proliferation, invasion and migration of bladder carcinoma cells are inhibited, promoting cancer cell apoptosis and inhibiting the epithelial–mesenchymal transition (EMT) of tumor cells (Ye et al. 2019). ERRs can directly and/or indirectly affect the physiological and molecular characteristics of tumor Leydig cells via the formation of a microenvironment (Kotula-Balak et al. 2018). ERRα in endometrial carcinoma cells plays a critical role in TGF-β-induced EMT through cancer-stromal interactions (Yoriki et al. 2019).

Interestingly, ERRs have also beneficial effects on disease treatment in the context of tumorigenesis and development. ERRα can also serve as an activating transcription factor or a transcriptional repressor depending on the cellular microenvironment, thereby promoting or inhibiting tumor growth in breast cancer (Misawa and Inoue 2015). In some patients with TNBC, the high expression of ERRα is a biomarker of the patients’ response to tamoxifen and a favorable prognostic factor for tamoxifen treatment (Manna et al. 2016). The overexpression of ERRβ or ERRγ can inhibit the proliferation of prostate cancer cells, and some research findings indicate that the expression of ERRβ or ERRγ in prostate carcinoma is frequently diminished (Misawa and Inoue 2015). The ERRγ agonist DY131 suppresses cancer growth and inhibits the Wnt signaling pathway. ERRγ is a novel tumor inhibitor that can block Wnt signaling and is a potential therapeutic target for gastric carcinoma (Kang et al. 2018).

ERRs have significant physiological and pathological effects on bone tissue. ERRα plays a role in tumor bone metastasis, which can occur in up to 70% of patients with advanced breast cancer, and ERRα can play multiple roles to promote the invasion of bone tissue by primary tumors (Misawa and Inoue 2015). In bones, the effects of cholesterol, statins, and bisphosphonates on osteoclast formation require ERRα. Both cholesterol-induced bone loss and bisphosphonate-mediated protective effects are lost in an ERRα-knockout (KO) mouse model (Wei et al. 2016). ERRα regulates bone remodeling by controlling osteoclastogenesis, which is a necessary cell differentiation process for bone resorption. The deletion of ERRα disrupts osteoclast differentiation and inhibits bone resorption (Wan 2010).

Additionally, ERRα inhibits osteoblastic differentiation (Gallet and Vanacker 2010). Another study showed that ERRα-KO mice were resistant to bone loss, and compared with those of wild-type mice, the number and activity of osteoclasts remained unchanged, while the bone formation rate and the activity of osteoblasts increased (Zhang et al. 2016). Some studies mainly point to ERRα as a switch that represses the differentiation of precursor cells into the osteoblastic pathway while favoring the adipocytic pathway (Gallet and Vanacker 2010). ERRα-deficient mice exhibited mild increases in cancellous bone volume and the amount of bone surfaces covered with bone-forming osteoblasts, whereas bone marrow fat volume was decreased (Delhon et al. 2009).

Moreover, ERRγ negatively regulates osteoblast differentiation and bone formation (Jeong et al. 2009). Bone trabeculae in ERRγ^+/−^ heterozygous mice lacking the ERRγ gene were increased compared to those of control animals (Cardelli and Aubin 2014). However, ERRγ is strongly expressed in bone marrow-derived macrophages (BMMs), which are osteoblast precursors; ERRγ suppresses the formation of multinucleated osteoclasts and attenuates the induction of nuclear factor of activated T cells c1, which is a critical modulator of osteoclastogenesis (Kim et al. 2019a). Suppressing ERRα and/or ERRγ can boost bone formation and compensate for bone loss due to aging or estrogen deficiency (Carnesecchi and Vanacker 2016). These experiments have fully demonstrated that ERRα and ERRγ play significant roles in maintaining bone homeostasis.

### Functions of ERRs in the pathogenesis of osteoarthritis

**Fig. 2:**
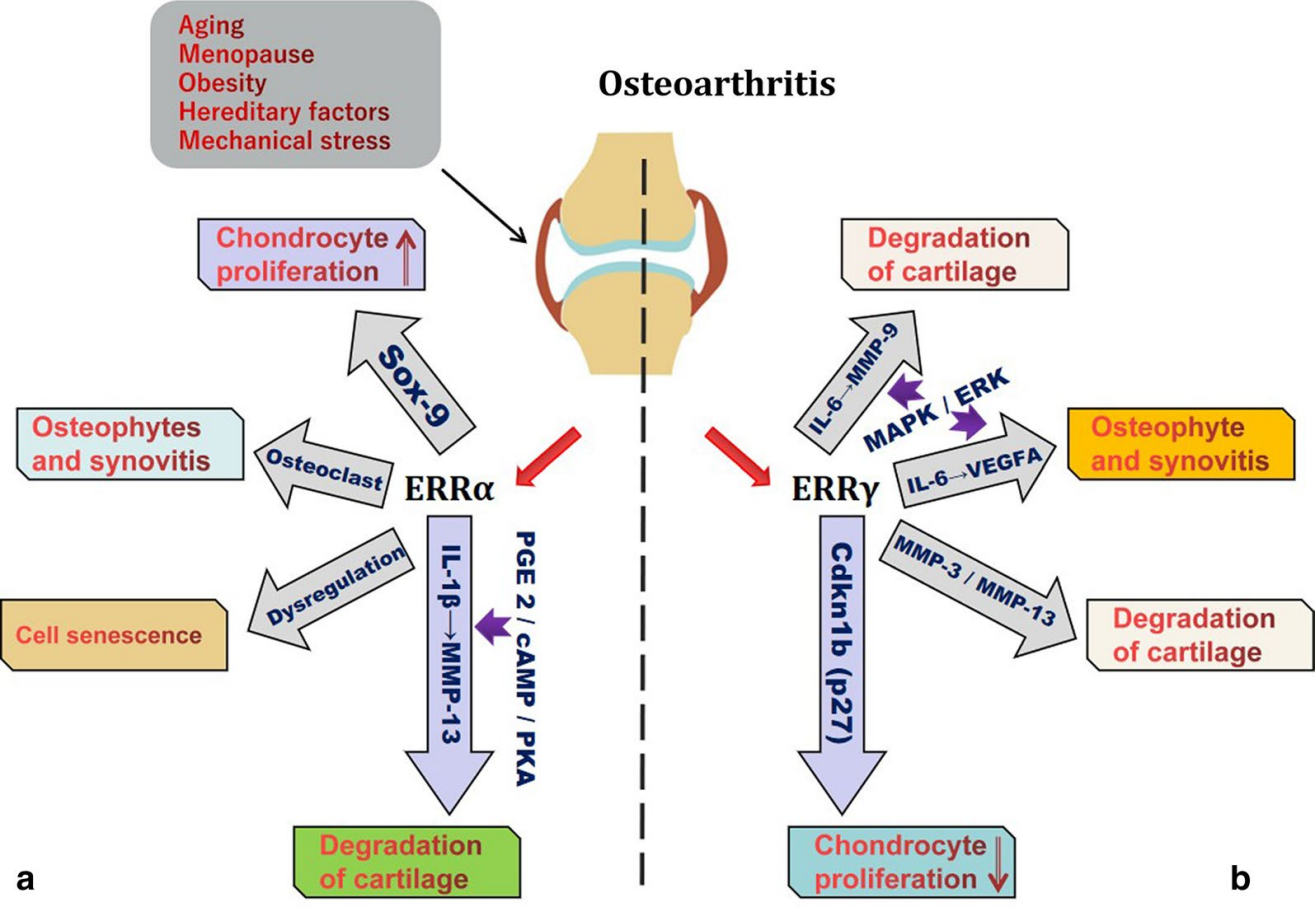
When the joint is affected by pathogenic factors such as aging, menopause, obesity, hereditary factors, and mechanical stress, the expression of the ERR family becomes disordered. **a** ERRα promotes chondrocyte proliferation by upregulating Sox-9. ERRα is generated in response to IL-1β stimulation through the PGE2/cAMP/PKA signaling pathways and regulates MMP-13 production. The dysregulation of ERRα may affect the aging of chondrocytes. ERRα participates in osteophyte formation and synovial hyperplasia by impacting osteoclasts. **b** The overexpression of the ERRγ, as regulators of IL-6, VEGFA and MMPs, will inevitably result in the dysfunction of molecular substances, disrupt homeostasis, and induce osteophyte, synovitis and cartilage degradation. ERRγ also restrains chondrocyte proliferation by upregulating p27

ERRs are highly expressed in the bone and cartilage tissue of the extremities and trunk, and they play essential roles in maintaining tissue homeostasis (Bonnelye et al. 1997; Bonnelye and Aubin 2005; Lorenzo 2017). Previous studies have shown that the ERRs, which are dominated by ERRα and ERRγ, are significantly associated with OA (Fig. 2).

### Estrogen-related receptor α

ERRα has dual functions in the occurrence and development of OA. On the one hand ERRα may response to the healing signal promotes the cartilage formation by upregulating Sry-type high-mobility-group box transcription factor 9 (Sox-9), but on the other, it can accelerate progression of OA in multiple ways (Bonnelye et al. 2011).

The effect of ERRα on cartilage formation is mainly associated with the regulation of its target gene of Sox-9 (Chen et al. 2014), which is involved in the proliferation, differentiation and maturation of chondrocytes. Sox-9 is a key regulator of chondrogenic differentiation and cartilage formation (Liu et al. 2018; Wu et al. 2018). ERRα directly or indirectly upregulates Sox-9 gene expression in chondrocytes (Bonnelye et al. 2007), thereby promoting the proliferation and accumulation of cartilage precursor cells and further induces the differentiation of these cells into mature chondrocytes; these cells participate in gristle formation, which plays an important role in maintaining the integrity of cartilaginous tissue (Bonnelye and Aubin 2005). The effect of ERRα on cartilage in vertebrate embryo development was verified in a zebrafish embryo model. In the zebrafish embryo model with ERRα gene knockout, the expression of Sox-9 was significantly lower than that in the equivalent wild-type embryo model during growth and development, which leads to malformation of the pharyngeal arch cartilage of the embryo in the course of upgrowth (Kim et al. 2015). Bonnelye et al. collected femoral condyles and tibial plateaus from OA patients after total knee arthroplasty to isolate and culture OA chondrocytes in vitro. OA chondrocytes were treated with the XCT790, a synthetic reverse agonist of ERRα, for 24 h, and the expression index of Sox-9 in OA chondrocytes was dose‐dependently downregulated by XCT790 (Kokabu et al. 2019). This experiment indirectly confirmed that ERRα slows OA chondrocyte loss by participating in cartilage formation.

The ERRα-mediated degradation of cartilage is associated with interleukin-1β (IL-1β) and matrix metalloproteinase-13 (MMP-13). IL-1β is an inflammatory cytokine that is closely related to the occurrence and development of OA (Liao et al. 2020; Wang and He 2018) and can participate in the pathological changes in OA through many mechanisms. When human OA chondrocytes were treated with IL-1β for 24 h, the expression of ERRα increased (Bonnelye et al. 2011). Further research showed that IL-1β stimulated ERRα expression via the PGE2/cAMP/PKA signaling pathway. MMP-13, also known as collagenase 3, performs a significant role in the process of OA (Li et al. 2017; Chan et al. 2017). MMP-13 can induce gristle damage by degrading collagens and proteoglycans in the cartilaginous extracellular matrix (ECM) (Fosang et al. 1996; Zhang et al. 2018a), and IL-1β is one of the major cytokines that induces MMP-13 expression (Tabeian et al. 2019). ERRα is generated in response to IL-1β stimulation through the PGE2/cAMP/PKA signaling pathways and is an important orphan nuclear receptor that regulates MMP-13 production. The level of IL-1-induced MMP-13 mRNA in OA chondrocytes was dose-dependently decreased by XCT790 (Bonnelye et al. 2011), which demonstrated that ERRα could upregulate the expression of MMP-13. ERRα is involved in IL-1β-mediated OA cartilage degradation and accelerates the progression of cartilage loss.

Osteocyte and chondrocyte senescence is one of the vital causes leading to the initiation and development of OA (Rahmati et al. 2017; Millerand et al. 2019). In chondrocytes in human OA, the expression of ERRα is dysregulated, and this condition becomes more common with age (Bonnelye and Aubin 2013), which indicates that ERRα may be a significant regulator of cellular senescence, thereby affecting the aging of chondrocytes (Huang et al. 2017).

ERRα is a crucial conditioning agent that promotes osteoclastogenesis and oxidative metabolism and is involved in many processes (Bae et al. 2017; Yang and Wan 2019), such as cell adhesion and transport, when expressed in osteoclasts (Bonnelye et al. 2010). ERRα is also expressed in highly motile cells such as macrophages and is an important regulator of biological functions (Leopold Wager et al. 2019). Macrophages lacking ERRα gene expression have decreased cell viability due to decreases in intracellular mitochondrial gene expression and reactive oxygen species (ROS) levels (Sonoda et al. 2007). A comprehensive analysis of the relationship between ERRα, osteoclasts and macrophages supports the role of ERRα in inflammatory diseases, such as OA and rheumatoid arthritis, that are associated with osteoclast-induced bone degradation leading to bone degeneration (Bonnelye et al. 2010). For example, enhanced subchondral bone remodeling and synovitis are the main pathological manifestations of osteoarthritis (Aho et al. 2017; Mathiessen and Conaghan 2017). The former is primarily characterized by macrophage infiltration and osteoclastogenesis (Zhu et al. 2019), while the latter is chiefly characterized by the infiltration of inflammatory cells such as macrophages and vascular proliferation (Zhang et al. 2020). Researchers verified the role of ERRα in osteoclasts in ERRα-KO mice and showed that ERRα deletion disrupted the expression of several major genes in cells, and ERRα-KO mice exhibited osteopetrosis due to osteoclast defects and decreased bone resorption, suggesting that ERRα may be a significant regulator of osteoclastogenesis (Yang and Wan 2019). However, while ERRα may participate in the occurrence and development of OA by regulating osteoclasts and macrophages, sufficient experimental evidence is required to elucidate its specific mechanism of action.

### Estrogen-related receptor γ

ERRγ can upregulate matrix metalloproteinase-9 (MMP-9) expression via the IL-6-mediated MAPK/ERK pathway and thus has an essential role in the destruction of OA cartilage (Son et al. 2017). An OA mouse model was used to show that the expression of ERRγ in cartilage was significantly higher than that in wild-type mouse cartilage. When ERRγ expression was inhibited by ERR siRNA or GSK5182, a reverse agonist of ERRγ (Kim et al. 2016), to inhibit its transcriptional activity, the expression of MMP-9 decreased when chondrocytes were stimulated with IL-6 (Son et al. 2017). It has been demonstrated that in OA chondrocytes, IL-6 mediates ECM degradation by stimulating the expression of MMP-9, and the overexpression of ERRγ amplifies this effect.

ERRγ can participate in the occurrence and development of OA through vascular endothelial growth factor A1 (VEGFA). IL-6 can stimulate chondrocytes to produce angiogenic factors, such as VEGFA (Son et al. 2017). The expression of VEGFA in OA is significantly related to the severity and pain intensity in OA (Hamilton et al. 2016; Guan et al. 2020), which is also crucial for osteophyte development (Wang et al. 2017b). After osteophytes are formed, the continuous production of VEGFA further stimulates vascular proliferation by osteophytes (Hashimoto et al. 2002). Another function of VEGFA is to stimulate the proliferation of OA synovium and synovial blood vessels, leading to inflammatory cell infiltration of the synovium and pain in patients with OA (Semerano et al. 2016). IL-6 is an important cytokine that regulates VEGFA gene expression (Kayakabe et al. 2012). ERRγ can regulate VEGFA gene expression by participating in the MAPK/ERK signaling pathway downstream of IL-6. Animal experiments indicated that the expression level of VEGFA in OA chondrocytes was reduced after using ERR siRNA to inhibit ERRγ expression or GSK5182 to inhibit ERRγ transcription (Son et al. 2017). ERRγ affects the expression of MMP-9 and VEGFA through the MAPK/ERK signaling pathway in chondrocytes, thereby participating in pathological processes such as cartilage degradation, vascular proliferation, osteophyte formation and synovial hyperplasia in the course of OA.

Experimental mouse models of OA were established by destabilization of the medial meniscus (DMM) surgery (Hong et al. 2020). Eight weeks later, the expression of ERRγ in the cartilage of OA mice was significantly increased compared with that in wild-type mice (Zhao et al. 2019), while the expression of matrix metalloproteinase-3 (MMP-3) and MMP-13 increased, and DMM mice showed significant OA manifestations, including cartilage erosion, subchondral osteosclerosis and osteophyte formation (Fang et al. 2018; Tetlow et al. 2001). When the experimental mice were replaced with Esrrg^+/−^ heterozygous mice lacking one Esrrg allele, the OA mouse model was then established by DMM surgery. Eight weeks later, the researchers examined heterozygous mice for the lack of an allelomorph of Esrrg, and the expression level of ERRγ in joint tissue was reduced (Son and Chun 2018), the expression levels of MMP-3 and MMP-13 induced by DMM were dramatically reduced, and the OA symptoms of cartilage erosion, subchondral osteosclerosis and osteophyte formation were significantly decreased compared with those of normal DMM mice (Zhao et al. 2019). This experiment shows that ERRγ upregulates the levels of MMP-3 and MMP-13 in articular chondrocytes via overexpression (Son and Chun 2018), which in turn leads to the degradation of collagens and proteoglycans in cartilage ECM and the exacerbation of OA symptoms (Guo et al. 2017; Hardy and Fernandez-Patron 2020). ERRγ is a novel regulator of the pathogenesis of osteoarthritis.

Transgenic technology was used to generate ERRγ-overexpressing mouse models, and the expression level of ERRγ in bone and cartilage was significantly higher than that in wild-type mice. Quantitative analysis of the proximal humerus, distal femur and proximal tibia revealed that the cartilage growth plate was obviously smaller than that of wild-type mice (Cardelli et al. 2013), and the declining regions were principally concentrated in the zone of proliferating cartilage (Michigami 2013). Compared with that of the control group, the height of the proliferating cartilage zone in the experimental group was decreased by 22%, and the primary reason for this dramatic decrease was that chondrocyte proliferation, differentiation, maturation and other behaviors were all affected (Cardelli et al. 2013; Hirota et al. 2018). Further studies demonstrated that the main trigger for the inhibition of chondrocyte proliferation ERRγ-mediated inhibition of chondrocyte proliferation by upregulating the expression of cyclin-dependent kinase inhibitor 1B (p27) (Kashiwagi et al. 2010), thus affecting the formation of cartilage growth plates. These studies show that ERRγ is a negative regulator of chondrocyte proliferation and differentiation, and its function appears to be opposite to that of ERRα (Bonnelye et al. 2011). In the occurrence and development of OA, the proliferation and differentiation of chondrocytes can slow the damage to articular cartilage (Charlier et al. 2019; Harrell et al. 2019). In summary, however, the expression of ERRγ in the cartilage of OA mice was significantly higher than that of wild-type mice (Zhao et al. 2019). Overexpression of ERRγ may affect the proliferation and differentiation of chondrocytes, which is detrimental to the process of self-repair in OA cartilage.

Bone and cartilage tissue homeostasis is coregulated by a variety of cytokines, growth factors and metalloproteases, and numerous molecular substances can synergetically or antagonistically maintain homeostasis (Mehana et al. 2019; Wojdasiewicz et al. 2014; Boehme and Rolauffs 2018). When bone and cartilage tissues are affected by pathogenic factors such as age, menopause, obesity, heredity, and mechanical stress, the expression of the ERR family, as regulators of IL-1/IL-6, Sox-9 and other molecular substances, becomes disordered (Loeser et al. 2012). Dysregulated expression will inevitably result in the dysfunction of cytokines, growth factors and metalloproteinases, disrupt homeostasis, and induce articular cartilage ECM degradation, synovial hyperplasia, osteophyte formation and other pathological manifestations in the occurrence and development of OA, as well as affect the duration of symptoms.

### ERRs and the development of innovative drugs

Because we generally lack a systematic and specific understanding of the pathogenesis of OA, the treatment options for early-stage patients are still limited (Dadabo et al. 2019; Murphy et al. 2016). Current OA treatment measures are symptomatic treatments but not etiological treatments. At best, these treatments can only alleviate pain and frequently fail to effectively curb the development of OA (Vinatier et al. 2016). Although joint replacement can ameliorate the condition of patients with advanced OA (Gademan et al. 2016), it is expensive, and artificial prostheses have limited service lives. In the case of postoperative complications such as prosthesis loosening and periprosthetic infection, revision surgery is required, which brings enormous psychological pressure and financial burden to patients (Kulshrestha et al. 2019; Schwartz et al. 2020). Based on the role of ERRs in the pathogenesis of osteoarthritis, the use of ERR molecular modulators to treat OA has a certain theoretical basis (Table 1) and important biomedical significance for the development of optimal therapies for the prevention and treatment of OA. Studies have shown that certain ERR-related inhibitors have good therapeutic effects on ERR-mediated diseases (Tripathi et al. 2020). For example, one ERRγ reverse agonist is a tetrasubstituted olefin analog that enhances the function of sodium iodide transporters in anaplastic thyroid cancer cells, thereby promoting the response to radioactive iodine treatment in vitro, and can be used as a potential therapeutic agent for ERRγ-mediated cancers (Kim et al. 2019b). Moreover, diethylstilbestrol (DES) can be used as a reverse agonist for all three ERR isoforms (Gibson and Saunders 2012; Greschik et al. 2002). Compound LingH2-10 is a novel selective inverse agonist of ERRα and can inhibit the growth of TNBC cells (Ning et al. 2017). Compound GSK5182 is an ERRγ inverse agonist (Zhang et al. 2018b; Kim et al. 2013) that can strengthen the antitumor efficacy of the tumor-reducing drug paclitaxel (Vernier et al. 2020). The compounds GSK 4716 and DY 131 are synthetic ERRβ/ERRγ agonists. These synthetic compounds have conspicuous inverse or positive agonistic effects on ERRs, and they may be used as effective drugs for the prevention and treatment of OA in the future. However, there are quite a few types of ERR inhibitors, their chemical structures and biological functions are exceedingly complex, and our understanding of their mechanisms of action is still far from complete. Because long-term use of DES increases the risk of malignant tumors in the reproductive system, this drug was restricted in 1971 (Huo et al. 2017; Titus et al. 2019; Smith et al. 2012). The compound XCT790 has always been regarded as a specific inverse agonist of ERRα, and it has been widely used in experiments related to ERRα (Kokabu et al. 2019). It is believed that XCT790 has the ability to disrupt the interaction between ERRα and PGC-1α and inhibit the growth of breast cancer cells. However, some studies suggest that XCT790 does not appear to be a very specific ERRα inverse agonist because at nanomolar concentrations, which is tenfold lower than the concentration required to inhibit ERRα, XCT790 is an effective, fast-acting mitochondrial uncoupler that enables rapid ATP depletion, and its effect is independent of ERRα inhibition (Vitto et al. 2019). Therefore, further research is needed to elucidate the mechanisms of action of ERR-related inhibitors on joints and other tissues to find innovative drugs to prevent and treat OA.

**Table 1:** Some small molecular modulators of estrogen-related receptors

Physiological effects	Molecular modulators	Targets	Notes	References
Inverse agonists	XCT790	ERRα	The detailed molecular mechanism of XCT790 binding to ERRα remains ambiguous	Kokabu et al. (2019), Vitto et al. (2019)
	LingH2-10	ERRα	IC_50_ = 0.64 ± 0.12 μM	Ning et al. (2017)
	Thiazolidinediones	ERRα		Patch et al. (2011)
	Statins	ERRα	Inhibiting effect in vivo; Inverse effect in vitro	Tripathi et al. (2020)
	DY40	ERRβ	The most potent ERRβ inverse agonist	Yu et al. (2017)
	GSK5182	ERRγ	Relatively non‐toxic with an oral LD_50_ in mice of greater than 1000 mg/kg	Joo et al. (2015)
	DY181	ERRγ	The most potent ERRγ inverse agonist; IC_50_ = 0.01 μM	Yu et al. (2017)
	Tetrasubstituted olefin analog	ERRγ		Kim et al. (2019b)
	4-OHT	ERRβ, ERRγ		Coward et al. (2001)
	TAM	ERRβ, ERRγ		Coward et al. (2001)
	DES	ERRα, ERRβ, ERRγ	Long-term use of DES can increase the risk of malignant tumors of the genital system	Lu et al. (2012)
Agonists	Cholesterol	ERRα	The detailed molecular mechanism of cholesterol binding with ERRα remains ambiguous	Casaburi et al. (2018); Li et al. (2019b)
	DY 131	ERRβ		Tiek et al. (2019)
	GSK4716	ERRγ	A potent ERRγ agonist with excellent selectivity over ERRα and ERRβ	Kim et al. (2009)
	Flavone and isoflavone	ERRα, ERRβ		Suetsugi et al. (2003)
	GSK9089	ERRβ, ERRγ		Zuercher et al. (2005)

## Conclusion

ERRα and ERRγ, which are typical orphan nuclear receptors, can regulate inflammatory cytokines and growth factors and thus exert significant effects on the occurrence and development of OA. Through the progress of basic experimental research, the roles of ERRs in OA have become clearer, while their mechanisms of action still require further study. More research on ERRs in osteoarthritis will provide an additional scientific basis for thoroughly understanding the pathogenesis of OA. The ultimate purpose is to identify drugs to prevent and treat movement system diseases such as OA based on the regulatory actions of ERRs. It is believed that with in-depth research, the advancement of technology and the deep integration of biomedicine and clinical medicine, patients with arthritis will be offered safer and more effective therapies in the immediate future, and this knowledge will also help us to develop novel treatment strategies.

## Data Availability

All data are available through cited literature.

## Abbreviations

OA: Osteoarthritis
ERRs: Estrogen-related receptors
ERRα/Esrra: Estrogen-related receptor α
ERRβ/Esrrb: Estrogen-related receptor β
ERRγ/Esrrg: Estrogen-related receptor γ
ERα: Estrogen receptor α
ERβ: Estrogen receptor β
DBD: DNA-binding domain
LBD: Ligand-binding domain
AF-1: Activation domain-1
AF-2: Activation function-2
PGC1α: Peroxisome proliferator-activated receptor γ coactivator 1α
NCOR1: Nuclear receptor corepressor 1
BAT: Brown adipose tissue
HK2: Hexokinase 2
GAPDH: Glyceraldehyde phosphate dehydrogenase
ENO1: Enolase 1
WAT: White adipose tissue
UCP1: Uncoupling protein 1
ER-: Estrogen receptor-negative
BMMs: Bone marrow-derived macrophages
VEGF: Vascular endothelial growth factor
TNBC: Triple negative breast cancer
EMT: Epithelial–mesenchymal transition
TGF: Transforming growth factor
KO: Knockout
Sox-9: Sry-type high-mobility-group box transcription factor 9
IL-1β: Interleukin-1β
MMP-13: Matrix metalloproteinase-13
ECM: Extracellular matrix
ROS: Reactive oxygen species
IL-6: Interleukin-6
MMP-9: Matrix metalloproteinase-9
VEGFA: Vascular endothelial growth factor A1
DMM: Destabilization of the medial meniscus
MMP-3: Matrix metalloproteinase-3
p27: Cyclin-dependent kinase inhibitor 1B
DES: Diethylstilbestrol
4-OHT: 4-Hydroxytamoxifen
TAM: Tamoxifen
